# Initial pyrolysis mechanism and product formation of cellulose: An Experimental and Density functional theory(DFT) study

**DOI:** 10.1038/s41598-020-60095-2

**Published:** 2020-02-27

**Authors:** Qing Wang, Hao Song, Shuo Pan, Nanhang Dong, Xinmin Wang, Shipeng Sun

**Affiliations:** Engineering Research Centre of Oil Shale Comprehensive Utilization, Ministry of Education, Northeast Electric Power University, Jilin City, Jilin, 132012 China

**Keywords:** Bioinorganic chemistry, Density functional theory, Molecular dynamics, Quantum chemistry, Reaction mechanisms

## Abstract

In this paper, analytical pyrolyzer coupled with a gas chromatography–mass spectrometry set-up (Py-GC/MS) and density functional theory(DFT) theory was used to reveal the initial pyrolysis mechanism and product formation mechanism of cellulose pyrolysis. We demonstrated an experimentally benchmarked molecular simulation approach that delineates pyrolysis process of cellulose. Experimental results indicated that the cellulose pyrolysis products mostly incorporate levoglucosan (LG), glycolaldehyde (HAA), 5-hydroxyfurfural (5-HMF), and the like. The constituents of fast pyrolysis products of cellulose and cellobiose demonstrated the identical trend, although the contents of certain products are different. Laying the foundation of experimental analysis, the reaction pathways of four categories of cellulose pyrolysis were outlined using DFT theory; the pathways are those of generating LG, HAA, and 5-HMF and the dehydration reaction in the process of cellulose pyrolysis. Also, by comparing the energy barriers of various reactions, the optimal pathway of different reactions were summarized. The deduced cellulose pyrolysis reaction pathway opened up new ideas for studying the pyrolysis behavior of cellulose.

## Introduction

Biomass is a clean renewable fuel source, and energy from biomass will definitely play a significant role in new energy systems in the future^1^. Pyrolysis is an important method of thermochemical conversion, which converts biomass into bio-oil and has the advantages of easy storage, transportation and high energy density. Therefore, biomass pyrolysis technology is considered to be a promising approach to the use of biomass energy. Biomass is a complex material that is mainly composed of crosslinked hemicellulose, cellulose, and lignin, along with extracts (tannins, fatty acids, and resins) and inorganic salts^2^. Numerous studies have indicated that cellulose is the most abundant organic linear polymer^3^. It is composed of D-glucose as a basic unit and is linked via β-O-4 glycosidic bonds. First, there are two controversial perspectives with respect to cellulose crystallinity. One contends that cellulose has a strong crystal structure^4,5^, whereas the other^6^ insists that cellulose has both crystalline and amorphous regions, depending on the periodic or random distribution of microfibers in the cellulose. Second, Each glucose monomer has six carbon atoms, two of which are attached to the glycosidic bond, and the other four are each attached to a hydroxyl group^7^. Previous research stated briefly that there are more active hydrogen bonds on glucopyranose^8^. These are some of the main features of cellulose. Because cellulose is the main component of biomass, studying the pyrolysis mechanism of cellulose contributes to understanding the pyrolysis law of biomass, which lays a foundation for developing the biomass pyrolysis process and the effective utilization of energy from biomass.

In depth studying the pyrolysis mechanism of cellulose is crucial to further understanding the thermochemical transformation of biomass. Previously, researchers proposed multifarious reaction kinetic models. Broido and Shafizadeh put forward the “B-S” model and proposed the theory that cellulose depolymerizes via heat to form active cellulose and that two kinds of parallel reactions occur. The earliest reaction has been recognized to be authoritative. Bradbury^9^ *et al*. considered that cellulose macromolecules undergo intermediate physical and chemical changes, such as vitrification^10^ or depolymerization^11^ to degree of polymerization(DP) approximate 200, and then it is transformed into designated products. This is in line with the B-S model and has been studied in more depth. Previous studies on cellulose pyrolysis have reported that the chemical and physical details of the B-S model are insufficient. Products, including LG, HAA, acetol, and formic acid, are also produced during cellulose pyrolysis^12^. It is widely recognized that cellulose is depolymerized via heat to make active cellulose, whereas the subsequent reaction step of activating cellulose remains controversial. Hence, Mamleev^13^ *et al*. proposed an improved model of the pyrolysis of cellulose that built on the previous model; the improved model clearly showed that there were two competing reactions in the pyrolysis process of cellulose. One reaction includes an intermediate that is formed via the E1 elimination reaction, and the other reaction produces LG and cellobiosan via transglycosylation. Furthermore, Bridgwater deduced that active cellulose is further broken into intermediates via dehydration and aromatization, resulting in the dehydration and condensation of coke and light gases through side chain radicals^14^. Some scholars have maintained that the production of light gases is indirectly related to the low temperature step or to anhydrous cellulose^15^. These kinetics models have largely simplified the complexity of the primary and secondary reactions of cellulose pyrolysis.

To support the simulation study, experiments have been proceeded for investigation of the cellulose pyrolysis. A thermogravimetric analyzer coupled with a Fourier-transform infrared spectrometer(TG-FTIR) has recently been developed and customized for pyrolysis research of the three components (cellulose, hemicellulose, lignin). For instance, Liu^16^ *et al*. studied the weight loss of cellulose and the evolution of typical functional groups. Using a summative law for the TG results, Biagini^17^ *et al*. obtained chemical composition of the biomass and predicted infrared spectrum of the volatiles. Py-GC/MS is another method widely applied to analyze the main components in the bio-oil that is produced in cellulose pyrolysis. Wang^18^ *et al*. have used Py-GC/MS to analyze the composition of the bio-oil in rapid pyrolysis of cellulose, by which a more refined cellulose pyrolysis model was established. Their work suggested that the remaining AC debris is rapidly pyrolyzed to form HAA and 1-hydroxy-2-propanone as well as secondary cracking gas and tar. In addition, related technology, such as scanning electron microscopy (SEM), X-ray diffraction (XRD) spectroscopy, Fourier transform infrared (FT-IR) spectroscopy, X-ray photoelectron(XPS) spectroscopy and isotope labeling method have also been performed to study the composition of small molecule products in cellulose pyrolysis^19–22^. While experiments provided valuable insight, the complexity of cellulose pyrolysis make the reaction mechanism remains unclear. During the rapid pyrolysis process, hundreds of parallel or continuous pyrolysis pathways occur, thereby forming complex liquid products, including water, dehydrated sugar and carbonyl groups, compounds, phenols, furans, cyclopentanone, linear esters, linear alcohols, oligomers, etc. It is difficult to analyze the detailed mechanism through experiments.

Density functional theory (DFT) has been widely used to study the chemical reaction during the pyrolysis of cellulose, which clearly indicates each molecular reaction at the atomic and molecular level, and has been confirmed by relevant experimental data. Therefore, some scholars^23–27^ employed theoretical methods to study the mechanism of pyrolysis and forcast possible reaction pathways. There has been extensive efforts made with respect to simulation calculations. These efforts have focused on the characteristics of the specific products in cellulose pyrolysis^28–30^. Dehydration reaction is the main reaction during the pyrolysis of cellulose, its formation mechanism has been widely investigated using density functional theory^31^. β-D-glucopyranose is the basic monomer of cellulose, and the monomer ring fractures during thermal cracking, which has also received more attention. In the pyrolysis reaction scheme proposed by Zhang^32^ *et al*., quantum chemical theory calculations reveal that the free radical mechanism has the highest energy barrier and the levoglucosan chain-end mechanism is the lowest. Zhang^33^ *et al*., found that various characteristic chains and dehydration units were generated from three internal configurations: the internal unit, the reducing end (RE end), and the non-reducing end (NR end)^34^. The generation mechanisms of small molecular weight products such as HAA, acetol, formic acid, acetic acid, furan, etc. have also been uncovered by density functional theory calculations and simulations^35–38^. Assary and Curtiss^39^ provided more details about retro-aldol reactions, which is a primary way to produce HAA. These results were confirmed by Zhang^39^ *et al*., who validated the important role of retro-aldol reactions in the HAA generation pathway. There has been extensive efforts on theoretical methods. However, there is a lack of integrated research on the mechanism of the initial stage of cellulose pyrolysis, a lack of comprehensive consideration of all possible pyrolysis reaction pathways, and research on these pathways to confirm the most favorable pathway. Hence, the mechanism of the initial stage of cellulose pyrolysis is still an unsolved mystery without consensus.

In this paper, rapidly pyrolyze of cellulose and cellubiose has been performed on the Py-GC/MS to investigate the distribution of cellulose pyrolysis products, enabling further analysis of products and chemical structures. Cellobiose(C_12_H_22_O_11_), a subunit of the biopolymer cellulose, has importance as a fundamental unit of structure in the field of plant structural sugars. This disaccharide can be obtained from the partial hydrolysis of cellulose resulting in β(1–4) linkage between the two d-glucopyranose residues. Cellobiose, therefore, serves as a good model compound for exploring β(1–4) glycosidic linkages^40,41^. Furthermore, on the basis of experiments, the density functional theory (DFT) was applied to calculate the designed reaction pathways of cellobiose, and the geometric shapes and thermal properties of all relevant structures of these pathways were compared to select the optimal pathway for the cellulose pyrolysis reaction. The deduced cellulose pyrolysis reaction pathway opens up new ideas for exploring the mechanism of how cellulose is converted into pyrolysis products on a microscale.

## Methods

### Experimental details

#### Materials

Microcrystalline cellulose Avicel PH101 was purchased from Sigma-Aldrich. This material is mainly extracted from fiber-rich plants. It is a white powder that has an average particle diameter of 50 μm, and the molecular formula is (C_6_H_10_O_5_)_n_. Microcrystalline cellulose can be converted into a component sponge. In addition, another studied chemical cellobiose was commercially available from Sigma-Aldrich; the molecular formula is C_12_H_22_O_11_, and it is a basic unit of cellulose that contains glucose monomers and glycosidic bonds. Before the experiment, the sample powder was dried in an oven at 100 °C for 2 h to remove absorbed water.

#### Experimental method

Cellulose and cellobiose were rapidly pyrolyzed in a Japanese Frontier EGA/PY-3030D multifunctional pyrolysis apparatus. The obtained gas phase product was analyzed using GC-MS. About 1 mg of sample was used for each experiment. The cellulose pyrolysis zone is in the range of 300–550 °C, and the maximum weight loss interval is 450–500 °C, producing plentiful volatile products^42^. It is worth noting that the high heating rate of the pyrolysis device and the poor thermal conductivity of cellulose leads to a temperature lag of about 100 °C^43^. Therefore, the pyrolysis temperature was set as 600 °C, and this was an appropriate condition for detecting the distribution of cellulose and cellobiose pyrolysis products. In order to observe the relationship between the experimental temperatures and the product contents, the pyrolysis temperatures were set to 400 °C, 500 °C, and 600 °C, respectively.

The pyrolysis vapor released in the collector was quickly analyzed using a PerkinElmer Clarus SQ8 GC-MS. Furthermore, the temperature of the injector must be maintained at 250 °C, and the cleavage product was brought into the chromatogram using high purity helium (99.999%) as a carrier gas with a split ratio of 1:20. The column temperature of the GC/MS was programmed to increase from 50 °C (where it was held for 5 min) to 250 °C at a heating rate of 10 °C/min. Finally, the chromatographic peaks of cellulose and cellobiose were identified with reference to the NIST MS library.

### Simulation details

#### Simulation model

**Figure 1:**
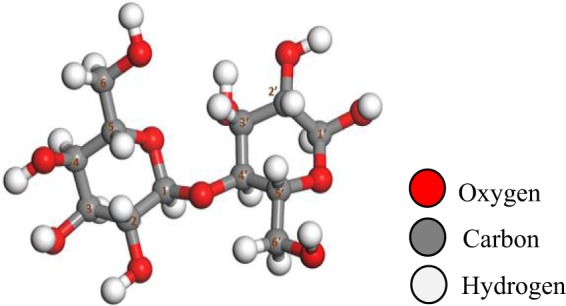
Optimized structure of the model compound cellobiose.

Cellulose is a macromolecular polymer that is linked to a glucose monomer as a basic unit that incorporates a β-O-4 glycosidic bond. To reduce computation time and computational costs, the pyrolytic properties of cellulose model compounds were investigated to replace the cellulosic structures. Early works that studied cellulose pyrolysis used glucose (cellulose monomer) as a model. The pyrolysis characteristics of glucose reflect the pyrolysis process of cellulose, but it does not consider the β-O-4 glycosidic bond of the glucose monomer. Because of the highly reactive glycosidic linkages, cellobiose is more susceptible than cellulose and glucose to cleavage at higher temperatures. Combined with the experience from previous studies, cellobiose was selected as the model, and the distribution of pyrolysis products was detected using Py-GC/MS. The kinetics model of cellulose pyrolysis was established to understand the main reaction mechanism of cellulose pyrolysis. As shown in Fig. 1, cellobiose is the fundamental cyclic unit of cellulose; it contains all of the chemical components present in cellulose.

#### Computational method

All of the calculations in this article were done using Accelrys’s Materials Studio DMol3. The dual value plus d function basis set (DND) was chosen, and the energies associated with the reactant (R), product (P), and transition state (TS) were calculated using a modified functional gradient approximation (GGA) function in the Review-Perdew-Burke-Ernzerhof (RPBE) functional. The convergence criteria for energy, force, and displacement were 2 × 10^−5^ Hartree (Ha), 0.004 Ha^−1^, and 0.005 Å, respectively. In addition, the SCF tolerance was 1*10^−5^ Ha. A number of scholars have pointed out the synergistic mechanism of cellulose in the rapid pyrolysis process, and hence, all of the reactions in this paper are based on synergistic reactions.

#### Pathway designed

**Figure 2:**
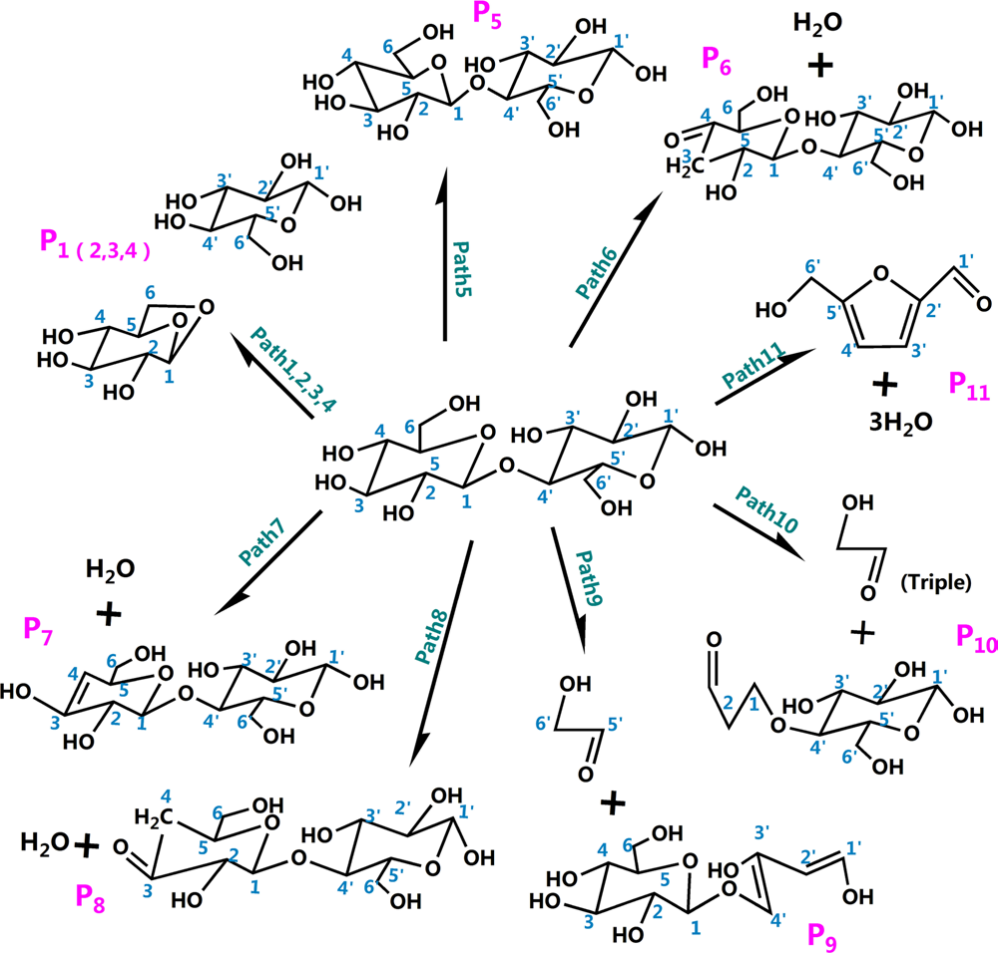
Reaction pathway of cellobiose pyrolysis.

The literature has mostly concentrated on explaining the initial mechanisms of cellulose pyrolysis, particularly the depolymerization of cellulose chains and the configuration of various small molecule products. Cellulose pyrolysis mainly produces three substances: (1) furans, (2) pyrans, and (3) linear small molecules. The foremost pyrolysis products include levoglucosan (LG), glycolaldehyde (HAA), and 5-hydroxymethyl-furan (5-HMF). The dehydration reaction that is caused by the carbonization of cellulose molecules with increased pyrolysis temperature is also an indispensable reaction. On the basis of the related theories of cellulose pyrolysis that have been previously published along with experimental research and simulation calculations, 11 reaction pathways that may occur during the thermal reaction of cellobiose are proposed. Figure 2 shows four kinds of reaction pathways of cellobiose pyrolysis, including the reactions that generate LG, HAA, 5-HMF, and dehydration reaction. Pathways 1–4 are the formation pathways of levoglucosan in cellulose pyrolysis. Among several predominant cellulose pyrolysis products^44^, LG is an authority pyrolysis primary product^45,46^; it is also an intermediate for the formation of other products^47,48^. Pathways 5–8 are process in which a hydroxyl group on a different carbon atom reacts with H to form a water molecule and then undergoes dehydration to generate a new product. Biomass pyrolysis experiments^49,50^ indicate that dehydration mainly occurs in the preliminary stages of biomass pyrolysis. Pathways 9 and 10 lead to reactants via dehydration, cleavage, and isomeric formation of glycolaldehyde HAA. Piskorz^51^ *et al*. believed that the two carbon fragments that are generated via the cleavage of cellulose monomers during pyrolysis are converted to HAA. As one of the main products of cellulose pyrolysis, 5-HMF is produced via three dehydration steps of hexose on the whole. The reactants in pathway 11 undergo repeated dehydration via the fructofuranose-intermediate-mechanism to form 5-HMF, which undergoes three basic reactions of ring opening, ring formation, and dehydration.

## Results and Discussion

### Pyrolysis product analysis

Birot^52^ showed that cellulose and cellobiose appear to have similar thermogravimetric weight loss curves. The maximum weight loss rate reached 80% when the pyrolysis temperature was 400 °C. Table 1 lists the main products of the rapid pyrolysis of cellulose and cellobiose. Table 1 shows that when cellulose and cellobiose were pyrolyzed at 400 °C, 500 °C, and 600 °C respectively, the distribution of various products during the pyrolysis is consistent, but the content of the products is disparate. At different temperatures, the content of various products and the temperature did not show obvious rules. On the basis of experimental results, all of the products can be classified into three types: pyrans (LG), furans (5-HMF, furfural), and linear small molecule compounds (CO_2_, acetaldehyde, acetic acid, etc.). Because the furan ring is more stable than the pyran ring, there tends to be more furan than pyran produced during pyrolysis^53^. During pyrolysis of cellulose, the pyran ring may be thermally broken to generate an intermediate, and the intermediate is further cyclized to form a furan ring; the small molecule product is then further cleaved to form HAA, propionic acid, acetaldehyde, and the like. LG has the highest content of the product in the pyrolysis process, and the content of LG is quite diverse in cellulose and cellobiose; this is consistent with the theory that the production of LG is related to the degree of polymerization of the cellulose chains. The high degree of polymerization results in higher content of LG in cellulose than in cellobiose. Remarkably, in cellobiose, the content of furan substances (such as 5-HMF and furfural) is higher than in cellulose. The yield of small molecule products in pyrolysis of cellobiose is higher than that produced by pyrolysis of cellulose. The experimental results reveal that LG and 5-HMF in cellulose pyrolysis products are significant products. Hence, the simulation part of this paper focuses on the production process and reaction energy barrier.

**Table 1:** Main products of rapid pyrolysis of cellulose and cellobiose (% in relative area).

Residue Time (min)	Compound	Formula	400 °C		500 °C		600 °C	
			Cellulose	Cellubiose	Cellulose	Cellubiose	Cellulose	Cellubiose
3.03	Carbon dioxide	CO_2_	1.204	3.138	2.436	2.370	1.67	4.08
3.15	Acetaldehyde	C_2_H_4_O	0.618	0.182	0.283	0.432	0.39	0.81
3.41	1-Propen-2-ol, acetate	C_5_H_8_O_2_	0.589	0.438	1.256	1.451	1.72	2.12
3.81	Methacrolein	C_4_H_6_O	0.003	0.295	0.088	0.142	0.33	0.2
3.95	Acetic acid ethenyl ester	C_4_H_6_O_2_	0.006	0.434	0.139	0.302	0.63	0.68
4.09	Furan, 3-methyl-	C_5_H_6_O	0.050	0.407	0.237	0.414	0.22	0.37
4.22	Acetic acid	C_2_H_4_O_2_	0.032	0.519	0.201	0.614	0.17	0.66
4.95	2-Propanone, 1-hydroxy-	C_3_H_6_O_2_	0.0409	0.295	0.393	0.056	0.5	1.18
5.43	2,3-Pentanedione	C_5_H_8_O_2_	0.002	0.0690	0.0218	0.019	0.05	0.1
6.03	2-Vinylfuran	C_6_H_6_O	0.034	0.095	0.103	0.099	0.03	0.17
7.23	2(5H)-Furanone	C_4_H_4_O_2_	0.034	1.404	0.056	2.030	0.05	0.26
7.66	Propanoic acid, 2-oxo-, methyl ester	C_4_H_6_O_3_	0.037	0.265	0.175	0.443	0.17	0.62
8.38	3-Furaldehyde	C_5_H_4_O_2_	0.026	0.023	0.044	0.068	0.06	0.06
8.91	Furfural	C_5_H_4_O_2_	0.777	4.207	1.098	5.4245	0.95	4.16
9.06	2-Amino-1,3,5-triazine	C_3_H_4_N_4_	0.015	0.150	0.078	0.216	0.03	0.33
9.73	2-Propanone, 1-(acetyloxy)-	C_5_H_8_O_3_	0.139	0.368	0.189	0.266	0.13	0.43
11.49	1,2-Cyclopentanedione	C_8_H_12_O_2_	0.304	0.655	0.532	0.104	0.63	1.21
11.73	2,5-Furandione, dihydro-3-methylene-	C_5_H_4_O_3_	0.0129	0.031	0.050	0.064	0.02	0.03
12.22	5-Methyl-furfural	C_6_H_6_O_2_	0.035	0.697	0.239	1.042	0.28	1.16
13.26	Oxazolidine, 2,2-diethyl-3-methyl-	C_8_H_17_NO	0.621	0.389	0.891	0.649	0.53	0.26
13.79	1,2-Cyclopentanedione, 3-methyl-	C_6_H_8_O_2_	0.091	0.230	0.226	0.484	0.46	0.47
15.00	2,5-Dimethyl-4-hydroxy-3(2H)-furanone	C_6_H_8_O_3_	0.013	0.213	0.732	1.083	0.52	0.58
15.61	Maltol	C_6_H_6_O_3_	0.014	0.139	0.127	0.198	0.13	0.14
16.47	4H-Pyran-4-one, 2,3-dihydro-3,5-dihydroxy-6-	C_6_H_8_O_4_	0.213	1.121	0.327	1.176	0.15	0.45
17.20	4H-Pyran-4-one, 3,5-dihydroxy-2-methyl-	C_6_H_6_O_4_	0.360	0.897	1.308	1.337	1.5	0.85
17.53	5-Hydroxymethylfurfural	C_6_H_6_O_3_	0.705	18.444	1.329	21.537	1.55	11.6
21.97	D-Allose	C_6_H_12_O_6_	0.588	2.488	2.083	4.931	8.2	1.25
23.84	Levoglucosan (LG)	C_6_H_10_O_5_	66.185	52.849	79.607	44.021	75.89	35.71
24.83	1,6-Anhydro-à-d-galactofuranose	C_6_H_10_O_5_	27.250	9.557	5.749	9.029	3.03	4.96

### Optimized geometries of reactant, transition state, and product

Before the molecular dynamics simulation, the reactants, intermediates, and products were geometrically optimized to search for the lowest energy points. To ensure the accuracy of the established cellulose pyrolysis model, the transition state of geometry optimization was determined using a TS search, and the identical basis set was used to calculate the reactant (R), intermediate (IM), product (P), and transition state (TS). Vibrational frequency analysis shows that the transition state has only one virtual frequency, whereas the reactants, intermediates, and products have no virtual frequencies. Structural parameter information of the optimized reactant, product, and transition state structure in each pathway are listed in Tables 2–7.

**Table 2:**
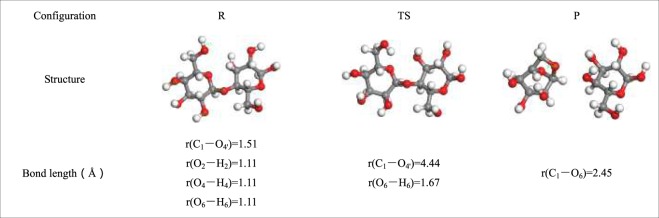
Optimized configuration in pathway 1.

**Table 3:**
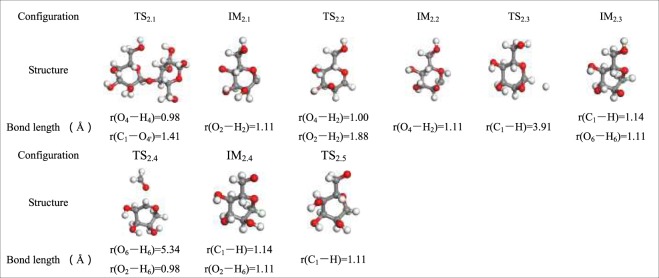
Optimized configuration in pathway 2.

**Table 4:**
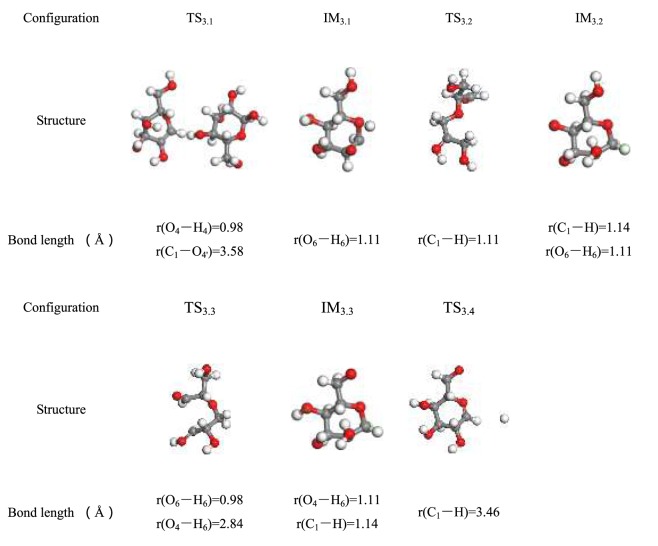
Optimized configuration in pathway 3.

**Table 5:**
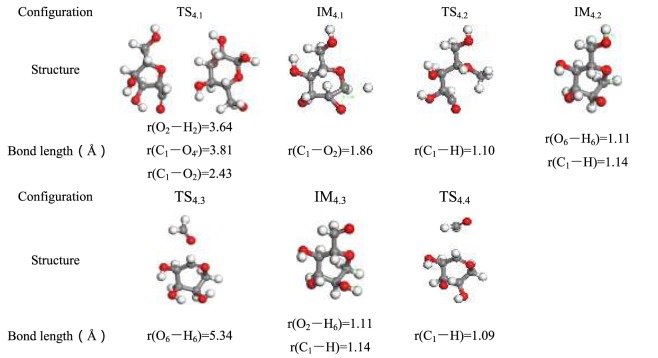
Optimized configuration in pathway 4.

**Table 6:**
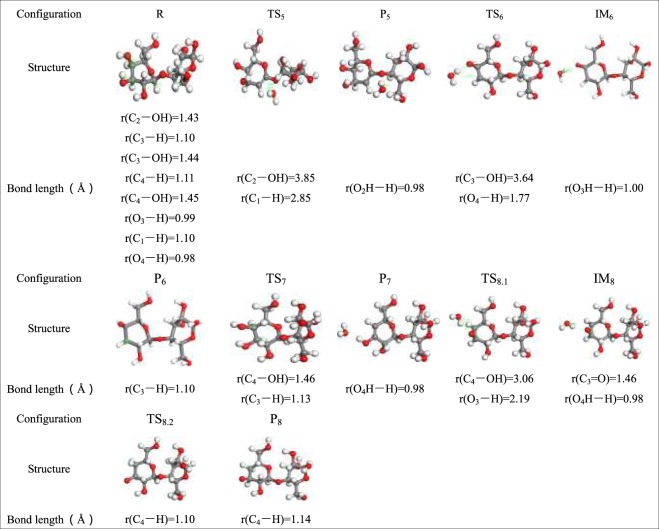
Optimized configuration in pathway 5–8.

**Table 7:**
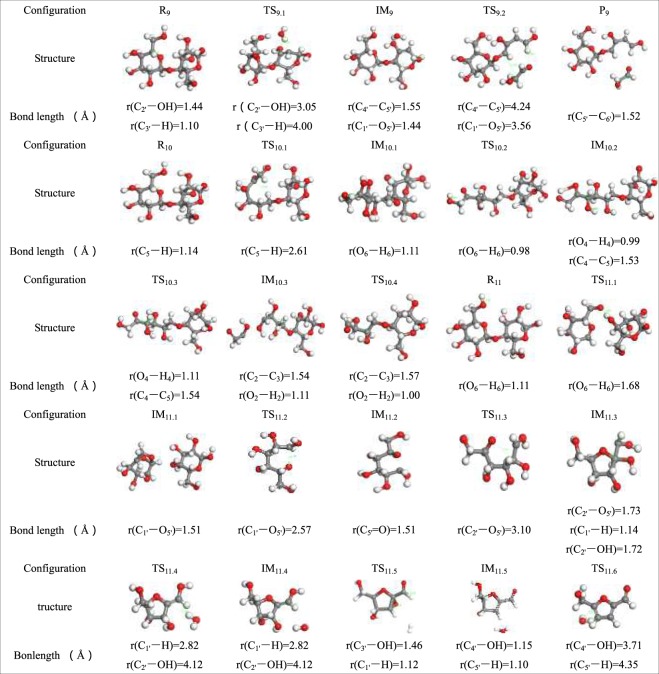
Optimized configuration in pathway 9–11.

### Thermodynamic analysis of pyrolysis reaction process

For the purpose of developing an in depth understanding of the energy transformation during the pyrolysis of cellulose, the thermodynamic parameters ΔH^Θ^ and ΔG^Θ^ of the optimized reactants and products of thermal cracking reactions at disparate pyrolysis temperatures (298, 500, 650, 800, and 950 K) were then calculated. The standard thermodynamic change in ΔH is the thermodynamic amount of the product minus the reactant, and the residual is zero-point corrected. The relevant thermodynamic parameters of the reactants and products in the 12 pathways at different reaction temperatures are summarized in Table 8. Analysis of the relationship between ΔH^Θ^ and temperature is shown in Table 8. It is known that the correlation between ΔH^θ^ and temperature is not closely related. Also, the ΔH^θ^ values of partial pathways increase with respect to temperature; nevertheless, the section barely changes during heating.

**Table 8:** Changes in standard thermodynamic parameters at different temperature.

Pathways	Parameters(KJ/mol)	298 K	500 K	650 K	800 K	950 K
Pathway1 Pathway2	ΔH^Ө^	−10.42	−12.61	−14.37	−16.3	−18.38
Pathway3 Pathway4	ΔG^Ө^	−11.78	−12.09	−11.7	−10.87	−9.68
Pathway5	ΔH^Ө^	−7.92	−6.05	−5.7	−5.95	−6.59
	ΔG^Ө^	−17.05	−23.856	−29.27	−34.69	−40.02
Pathway6	ΔH^Ө^	−49.43	−53.67	−57.67	−62.29	−67.41
	ΔG^Ө^	−46.46	−43.34	−39.71	−35.08	−29.53
Pathway7	ΔH^Ө^	−14.18	−15.34	−17.34	−19.99	−23.05
	ΔG^Ө^	−19.79	−23.38	−25.54	−27.15	−28.23
Pathway8	ΔH^Ө^	−51.19	−56.45	−61.01	−65.99	−71.29
	ΔG^Ө^	−46.28	−41.55	−36.46	−30.267	−23.11
Pathway9	ΔH^Ө^	−96.3	−98.91	−103.47	−109.73	−117.25
	ΔG^Ө^	−110.96	−120.38	−126.25	−130.86	−134.17
Pathway10	ΔH^Ө^	−261.52	−274.5	−289.83	−308.65	−330
	ΔG^Ө^	−253.24	−244.52	−233.53	−218.57	−199.83
Pathway11	ΔH^Ө^	−630.87	−690.43	−749.2	−816.54	−890.2
	ΔG^Ө^	−555.13	−488.59	−420.24	−337.23	−240.94

**Figure 3:**
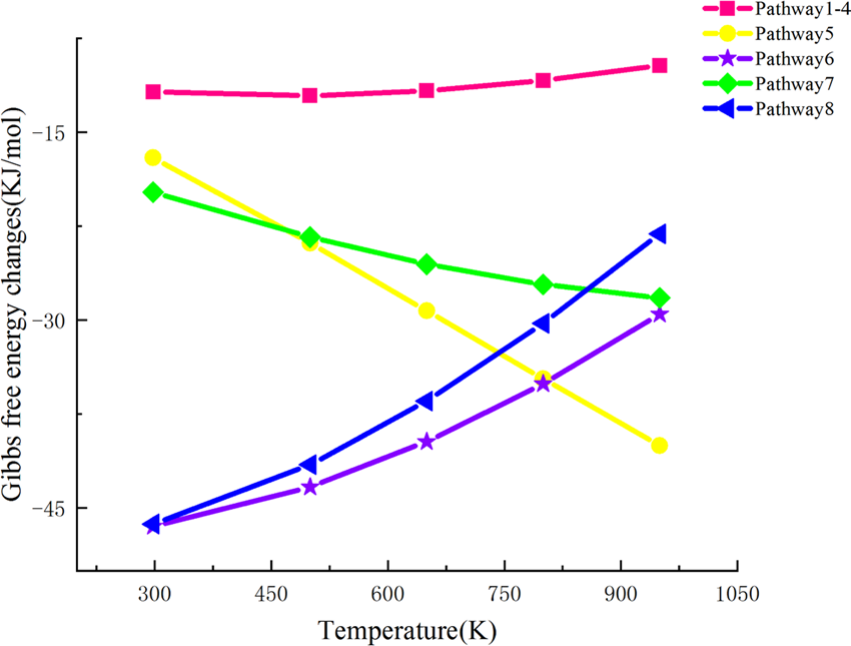
Gibbs free energy ΔG relationship diagram for pathway 1–8.

**Figure 4:**
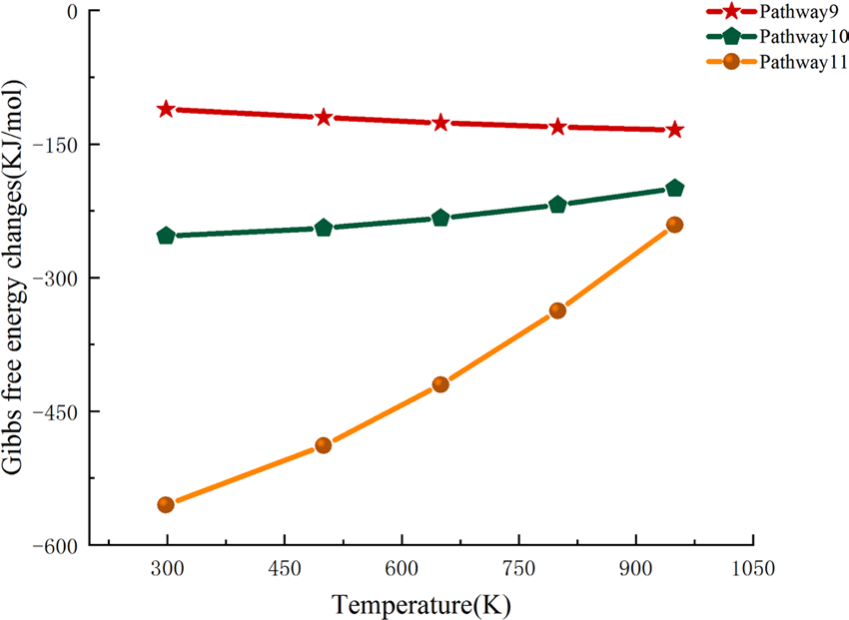
Gibbs free energy ΔG relationship diagram for pathway 9–11.

The Gibbs free energy (ΔG^Ө^) is also an essential thermodynamic parameter. ΔG^Ө^ is the thermodynamic correlation between reaction spontaneity and reactant conversion. When ΔG^θ^ < 0, a reaction can be carried out spontaneously. Figures 3 and 4 are graphs that show the relationship between ΔG^θ^ and the temperature of the 12 reaction pathways. From comprehensive analysis of Table 8 and Figs. 3 and 4, it can be inferred that as the pyrolysis temperature gradually increases, the ΔG^θ^ values of all of the reactions decrease, and this also explains the phenomenon that high temperature favors cellulose pyrolysis. When the temperature is lower than 500 K, the ΔG^θ^ values for pathways 1–4 are a relatively stable value. At this stage, the thermal effect is not significant because of the lower temperature, and this is consistent with the first stage of the thermogravimetric analysis of cellulose. When the temperature is higher than 500 K, ΔG^θ^ begins to decrease, and this indicates that when the temperature surpasses 500 K, the thermal decomposition of cellobiose produces LG. This is consistent with the theory obtained by the previous researchers who found that the main cellulose pyrolysis range is 550K–800K. It is said that the reactions between LG and HAA are competitive, and therefore, the thermodynamic quantitative values of pathways 1–4, which produce LG, are compared to pathways 10–11, which produce HAA. Because ΔH^Θ^_1–4_ < ΔH^Θ^_10–11_, pathways 10–11, which generate LG, are more likely to occur than pathways 1–4, which generate HAA. However, ΔG^θ^_1–4_ < ΔG^θ^_10–11_ indicates that the production of LG via pyrolysis is superior to the yield of HAA, and this corresponds to the theory that LG is the most significant product in the pyrolysis process of cellulose.

### Kinetic analysis of the pyrolysis process of each pathway

#### Generating pathways of main product LG

The activation energy values of the 12 reaction pathways in this work were determined based on the transition state theory. In light of transition state theory, the smallest energy discrepancy between the saddle point on the potential energy surface (i.e., the transition state) and the reactant represent the activation energy. In the following pathways, R represents a reactant, TS represents a transition state, IM represents an intermediate, and P represents a product. Moreover, in the intermediate Im a.b, a represents the number of pathways and b represents the order of object in the pathways. For instance, IM_2.1_ represents the first intermediate of the second pathway, and IM_3.2_ represents the second intermediate of the third pathway, and so on.

**Figure 5:**
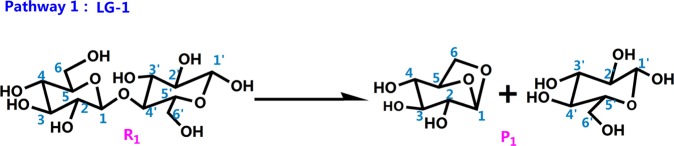
Pathway 1 reaction process.

The first pathway is the pathway of LG generated by the synergistic reaction of cellobiose summarized by the predecessors. Some scholars believed that this pathway is the optimal pathway. In consequence, this paper chosed this pathway as a comparison with other pathways that generated LG. Besides, in order to reduce the errors caused by the calculation basis set and parameter settings between the pathways that generated LG, all reaction pathways in this article were calculated using a unified basis set and calculation parameters. Under this premise, the obtained energy barrier of pathway 1 is more favorable for comparison with the energy barriers of other paths that generated LG in the paper. As shown in the Fig. 5, the H atom of the C_6_–OH hydroxyl group transfers to the glycosidic bond in cellobiose, and the glycosidic bond cooperatively cleaves. The bond length of r(C_1_–O_4′_) is 1.51 Å in R_1_, and this becomes 4.44 Å in TS_1_. At the same time, C_6_–O**·** connects to C_1_ to construct LG with a bond length r(C_1_–O_6_) of 2.45 Å. The activation energy of Pathway 1 is 327 kJ/mol, and this is slightly different from the value (377.54 kJ/mol) calculated by Huang *et al*. The differences in the model compound construction, dihedral angles, molecular bond lengths, and different basis functions lead to this difference.

**Figure 6:**
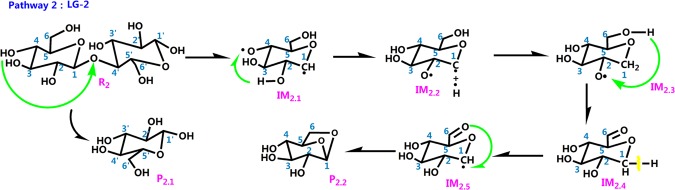
Pathway 2 reaction process.

Figure 6 shows the process by which cellobiose produces LG via pathway 2. The first step in the reaction is the simultaneous cleavage of the glycosidic bond and the H atom of –O_4_H to form intermediate IM_2.1_ which contains C_4_–O**·**. The IM_2.2_ is generated via the transfer of H atom from O_2_–H_2_ to C_4_–O**·**. The energy barrier of this reaction step is 140.64 kJ/mol, which illustrates that the reaction process of pathway 2 is an endothermic reaction. The length of O_6_–H_6_ increases from 1.11 Å in IM_2.3_ to 5.34 Å in IM_2.4_. The new bond C_6_=O is about to form when H_6_ shifts to C_2_–O**·** in IM_2.4_. The activation energy of the reaction step is as high as 449.66 kJ/mol, and this requires absorbing even more heat. Then, the H group that is initially bound to C_1_ is removed to compose IM_2.5_. At the end of the reaction, C_6_=O bonds to C_1_ to form the pyrolysis product P_2.2_ (LG). Pathway 2 follows the free-radical mechanism of LG, and the cellulose chain is first broken into an anhydroglucose group in the pyrolysis of cellulose; this is then converted to levoglucosan. Free radicals formed via homogeneous cleavage of cellulose chains.

**Figure 7:**
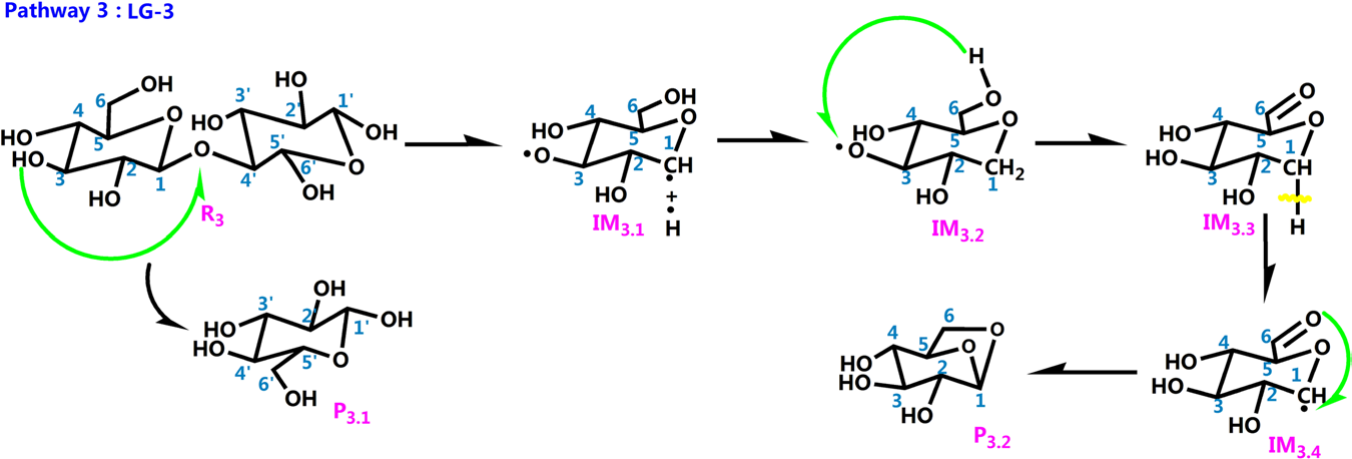
Pathway 3 reaction process.

Pathway 2 is similar to the first step of pathway 3, and the same is true for C_1_ and cleavage of the glycosidic bond. Figure 7 gives the reaction process of pathway 3 in cellulose pyrolysis. In the energy barrier diagram, the highest energy barrier in each pathway is shown. This indicates that this step is the rate-determining step for the whole levoglucosan formation process, which occurs via a free-radical mechanism. In the course of pyrolysis, a mass of free H groups concatenate to unsaturated C_1_ to form C_1_–H for IM_3.2_. Thereafter, H transfers from C_6_–OH to C_4_–O**·**, the bond O_4_–H_6_ lengthens to form TS_3.3_, and the length of O_4_–H_6_ shortens from 2.84 Å to 1.11 Å. These observations indicate that the transition state TS_3.3_ generates a new bond O_4_–H_6_ that is present in IM_3.3_. It can be refered to Fig. 7 that the IM_3.3_ reacts to P_3.2_ through breaking C_1_–H bond plus O_6_ shifts from C_6_ to C_1_ and forming O_6_–C_1_.

**Figure 8:**
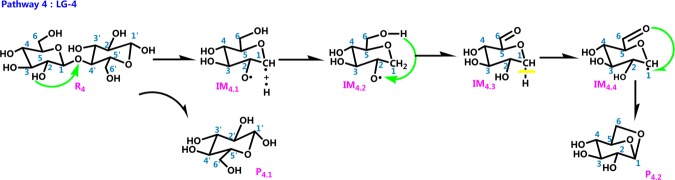
Pathway 4 reaction process.

Form Fig. 8, O_2_–H_2_ breaks as the pyrolysis temperature increases, glucopyranose forms in pathway 4. At the same time, consistent with the sharp decrease of r(C_1_–O_2_) from 2.43 Å to 1.86 Å, it can be inferred that the unsaturated O_2_ that lost an H atom then connects with C_1_. Heat in the amount of 226.15 kJ/mol is absorbed to form intermediate IM_4.1_. In the pyrolysis reaction, the H group combines with C_1_ to produce intermediate IM_4.2_, wherein the new bond r(C_1_–H) is 1.14 Å. The C_6_–OH breaks directly by adjacent H atoms transfer shown in Fig. 8. Specifically, the H_6_ atom moves from C_6_–OH to C_2_–O**·** to form a hydroxy group (O_2_–H_2_) and C_6_=O bond in IM_4.3_, respectively, through TS_4.3_; and O_6_ atom transfers from C_6_ to C_1_ to generate C_1_–O_6_ in P_4.2_, via TS_4.5_. The energy of the glycosidic bond cleavage in the first step of pathway 4 consumed less energy than pathways 2 and 3, and this is consistent with the energy of 155–228 kJ/mol that is obtained in the experiment^54^. This verifies the rationality of this step.

**Figure 9:**
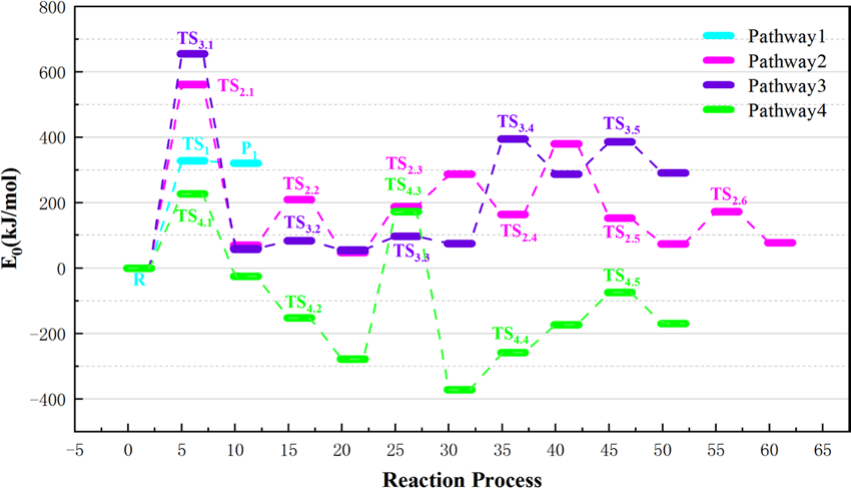
Reaction energy barrier diagram of pathway 1–4.

Analyzing the change in activation energy during each reaction enables us to develop further kinetic analysis. Figure 9 shows potential energy curves for pathways 1–4; the curves depict the changes in thermodynamic energy in each pyrolysis pathway that leads to LG. The energy barriers in the graph indicate the reaction energy of the transition state relative to that of the reactants, and the reaction energy is equal to difference between the product and reactant energy. In this article, there are four calculations about the pathway of LG generation. The first one is the pathway for synergistic reaction of cellobiose summarized by previous generations to generate LG. It is more convenient to compare with other energy barriers in the paper to generate LG. In this paper, the authors designed routes 2, 3, and 4 in strict accordance with the principle that H on different hydroxyl –OH groups was transferred to glycosidic bonds, which caused the glycosidic bonds to break. Combining with previous studies, the former usually only considered the cooperative cleavage of H and glycosidic bonds on the C_6_–OH hydroxyl group, while pathways 2, 3, and 4 in this article considered the reaction of H with glycosidic bond cleavage. In pathway 2, the H on the hydroxyl group of C_4_ was broken, and the H radical moved to the glycosidic bond to bind. Since the H radical moved far away, the heat absorbed during the reaction was as high as 560.81 kJ/mol, which was more difficult to achieve. Similar to Pathway 2, the free radical formed by the H cleavage on the hydroxyl group of C_3_ in Pathway 3 moved to the glycosidic bond far away, which caused a large amount of energy of 655.37 kJ/mol to be absorbed in this step reaction. Although the path design of pathways 2 and 3 was strictly based on the principle of the combination of H radicals that were shed from the hydroxyl groups in cellobiose and glycosidic bonds, the energy of the H radicals in pathways 2 and 3 to break from C_3_–OH and C_4_–OH was not favorable. Hence, the cellobiose does not react according to pathways 2, 3 during the actual reaction.The H radical shed from the hydroxyl group of C_2_ in the pathway 4 moved to the glycosidic bond, and absorbed 226.15 kJ/mol of heat, and this step is the rate determining step of the entire reaction pathway 4. As calculated by Assary^55^ *et al*., Zhang^56^ *et al*., the comparison of the results shows that the path design of path 4 is more reasonable, and cellobiose may reacts to generate LG according to this path. Compared with the rate-determining step of absorption of 327.47 kJ/mol heat in pathway 1, the rate-determining step of pathway 4 is 226.15 kJ/mol. It can be inferred that in the process of cellobiose cracking to generate LG, pathway 4 is a valid competing path for pathway 1.

#### Dehydration reaction pathway

Carbohydrates have a large amount of hydroxyl –OH and are the dominant component of biomass, and thus, dehydration during cellulose pyrolysis is inevitable. From previous experience, it is known that the 1,2 dehydration mechanism and 1,3 dehydration mechanism are the most pervasive, more so than in alcohol dehydration^57,58^. Some scholars^59^ have proposed the hydrogen bonding-assisted Grob fragmentation mechanism and hydrogen bond-assisted pyran ring recombination mechanism to understand the dehydration phenomenon in biomass. Regarding the choice of mechanism, this study used the most widespread mechanism for analyzing the dehydration reaction and its product distribution during cellulose pyrolysis.

**Figure 10:**
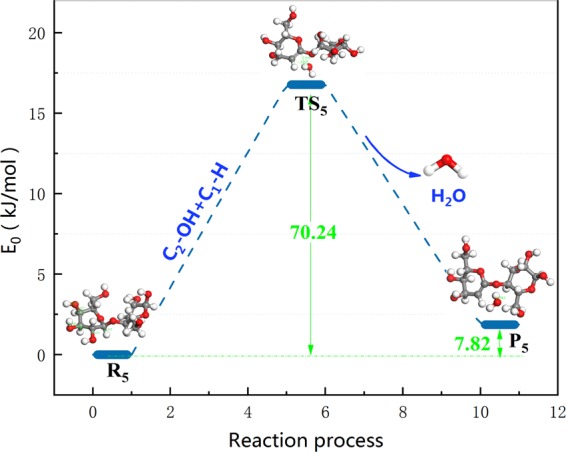
Reaction step and reaction energy barrier of pathway 5.

Pathway 5 abides by the 1,2 dehydration mechanism. The reaction step and reaction energy barrier of pathway 5 are summarized in Fig. 10. First, the H on C_1_ and the –OH on C_2_ are cleaved. Then, C_1_–H combines with C_2_–OH to generate water molecules, absorbing 70.24 kJ/mol of heat during the reaction, which existed imparity with the activation energy of 305.3 kJ/mol when glucopyranose was dehydrated that Zhang^60^ *et al*. Differences in the choice of model compounds and reaction conditions are the cause of differences in activation energy.

**Figure 11:**
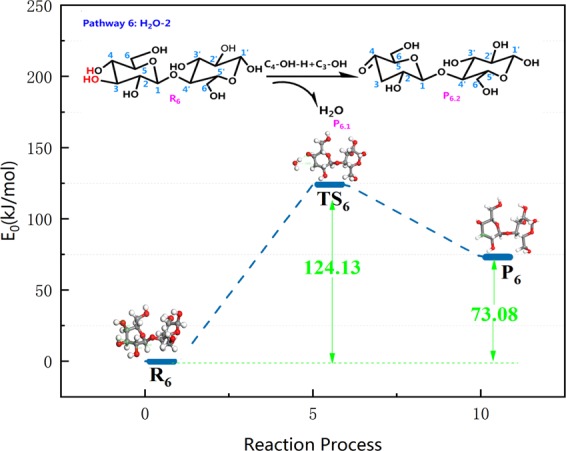
Reaction step and reaction energy barrier of pathway 6.

Paine III *et al*. proposed a hydrogen bond-assisted Grob fragmentation mechanism for carbohydrate pyrolysis. A similar mechanism can be derived from this. Also, hydrogen bonding assistance in the rearrangement of pinacol is another possible way for dehydration of –O_2_H in cellobiose. In pathway 6, the H of C_4_–OH combines with the dehydration of C_3_–OH shown in Fig. 11, and then the H on the hydroxyl–OH on C_4_ cleaves to generate P_6_ with a C_4_=O. With the release of 124.13 kJ/mol of heat, the activation energy of this reaction is lower than the other dehydration pathway in this paper. Therefore, it can be extrapolated that this pathway is the main dehydration process apart from pathway 5. The unsaturated C_3_ bond in R_6_ links to the free H radicals during pyrolysis to produce saturated P_6_.

**Figure 12:**
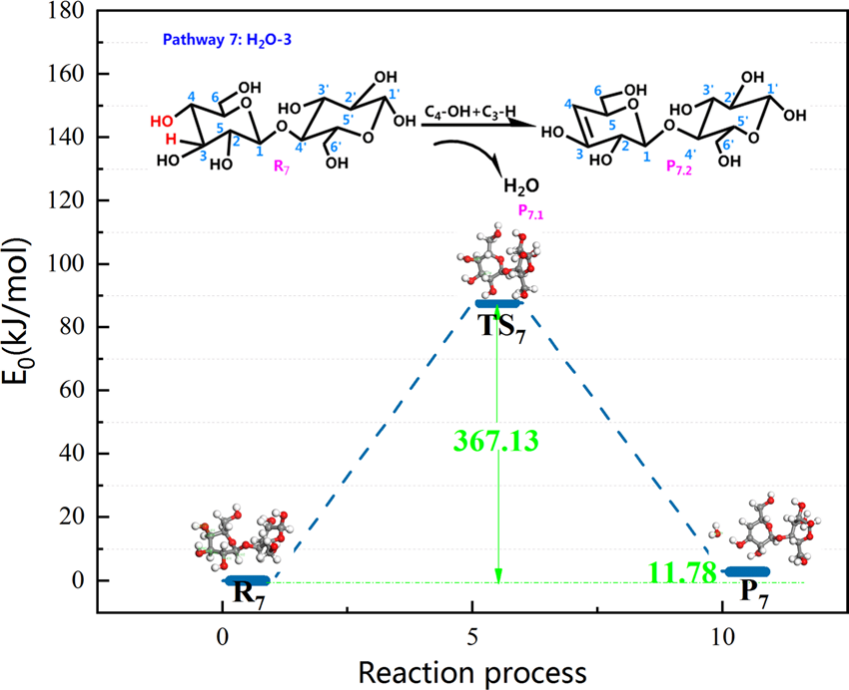
Reaction step and reaction energy barrier of pathway 7.

Figure 12 shows a overview of the pyrolysis process and the potential energy of cellobiose pathway 7. This pathway was a dehydration process with a lower energy barrier proposed by the predecessors. It is competitive in the pathway of cellulose dehydration reaction. Therefore, in this paper, the theoretical study of pathway 7 was carried out with the same base group and calculation parameters, which was beneficial to compare with the dehydration pathway designed by the author. It is conducive to select the optimal path for dehydration reaction. Pathway 7 displays 1,2-dehydration of C_4_–OH and C_3_–H, and the bond length of the participating reaction in TS_7_ merely changes by 0.03 Å compared to that in reactant R_7_. It can be deduced that the response rate of pathway 7 tends to be slow, and hence, there was no significant change in the bond length in TS_7_. Throughout the reaction shown in Fig. 12, 367.13 kJ/mol of heat is released. Nimilos^61^ *et al*. proposed that the 1,2 dehydration barrier of simple alcohols and LG is about 67–69 kcal/mol (280.46~288.83 kJ/mol); however, 367.13 kJ/mol of heat is released during the reaction that follows pathway 7. Obviously, there is a certain difference in the energy barrier. Fro, analysis, it is determined that the structure of cellobiose itself is more tangled than that of LG. The reaction structure is erratic when it goes through TS_7_ in the reaction, and the reaction rate is sluggish, which indirectly induces an increase in the reaction barrier. Thus, this causes the discrepancy in activation energy during the process. From the energy barrier figure, it can be inferred that H leaving C_3_ is the rate determining step of the pathway.

**Figure 13:**
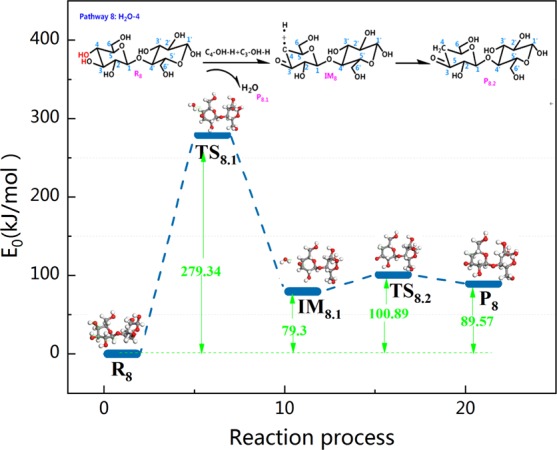
Reaction step and reaction energy barrier of pathway 8.

Taking into account the diversity of the reaction steps in pyrolysis of cellulose, pathway 8 is unlike the other dehydration pathways. The other dehydration reactions were synergistic reactions without intermediates, whereas pathway 8 leads to intermediate IM_8_. The reaction step and reaction energy barrier of pathway 8 are shown in Fig. 13. It then joins the H radical to form saturated P_8_. During the dehydration reaction of pathway 8, hydroxy –OH and H first detach from C_4_ and C_3_–OH, respectively. This releases 279.34 kJ/mol of heat while generating IM_8_, which has a C_3_=O double bond. Corresponding kinetic data have been reported with glucose as the model compound in work reported by Mayes *et al*. (E_a_, 304.8–336.2 kJ/mol vs 298.6–323.4 kJ/mol). In addition, the C_4_ of IM_8_ is not saturated, and therefore, the H radical that is produced in the pyrolysis process combines with C_4_ and eventually produces the saturated dehydration product P_8_.

Summarizing the previous experience, it can be inferred that the dehydration energy barrier during the pyrolysis of cellulose model compounds is 264.56–414.83 kJ/mol, and the dehydration energy barriers obtained by some studies were concentrated in 298.6–336.2 kJ/mol, which was closed to the energy barrier of pathway 7 (C_4_–OH + C_3_–H, 367.13 kJ/mol) and pathway 8 (C_4_–OH–H + C_3_–OH–H, 279.34 kJ/mol) calculated with density functional theory in this paper, further illustrates the rationality of the calculation results of pathways 7,8. However, the calculated energy barriers for pathway 5 (C_2_–OH + C_1_–H) and pathway 6 (C_4_–OH–H + C_3_–OH) in this paper were 70.24 kJ/mol and 124.13 kJ/mol, as compared with Lu *et al*. and Mayes *et al*., the calculation results of pathway 6 and 5 were more favorable in thermodynamics, which indicated that the dehydration reaction of pathway 5 and pathway 6 is more likely to occur in kinetics.

#### Small molecule product generation pathway

Glycolaldehyde HAA is a crucial pyrolysis product, and its yield is second only to LG in bio-oil. It is primarily derived from the cleavage of cellulose as a whole (ring breakage). Lu^62^ *et al*. considered that HAA is chiefly derived from the cleavage of C_1_–C_2_, C_5_–C_6_, C_3_–C_4_ in the cellulose monomer. Furthermore, a tiny fraction of HAA is generated from secondary cleavage of LG. Two reaction pathways for producing HAA are proposed in this paper, and the remarkable difference between pathways 10 and 9 is that the former incorporates a retro-diels-alder reaction.

**Figure 14:**
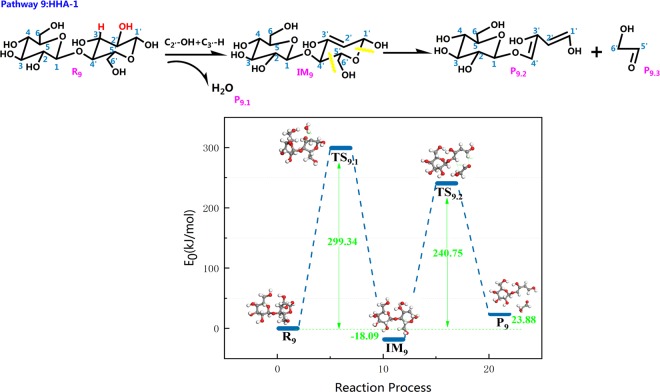
Reaction step and reaction energy barrier of pathway 9.

As shown in Fig. 14, pathway 9 first goes through dehydration of cellobiose, and then C_4′_–C_5′_ and C_1′_–O_5′_ of cellobiose are cleaved, leading to ring opening of the pyran ring. This results in the product P_9_, which has two C=C double bonds (C_1′_–C_2′_, C_5′_–C_6′_), and HAA molecules (two carbon fragments) and releases 240.75 kJ/mol of heat. According to Nimols *et al*., the energy barrier of the 1,2 dehydration process is in the range of 280–290 kJ/mol, and the calculation results in this paper are closed to this range. As seen from the combination of the reaction steps and the potential energy profile, the pyran ring that is subjected to dehydration is more likely to undergo the ring opening reaction.

**Figure 15:**
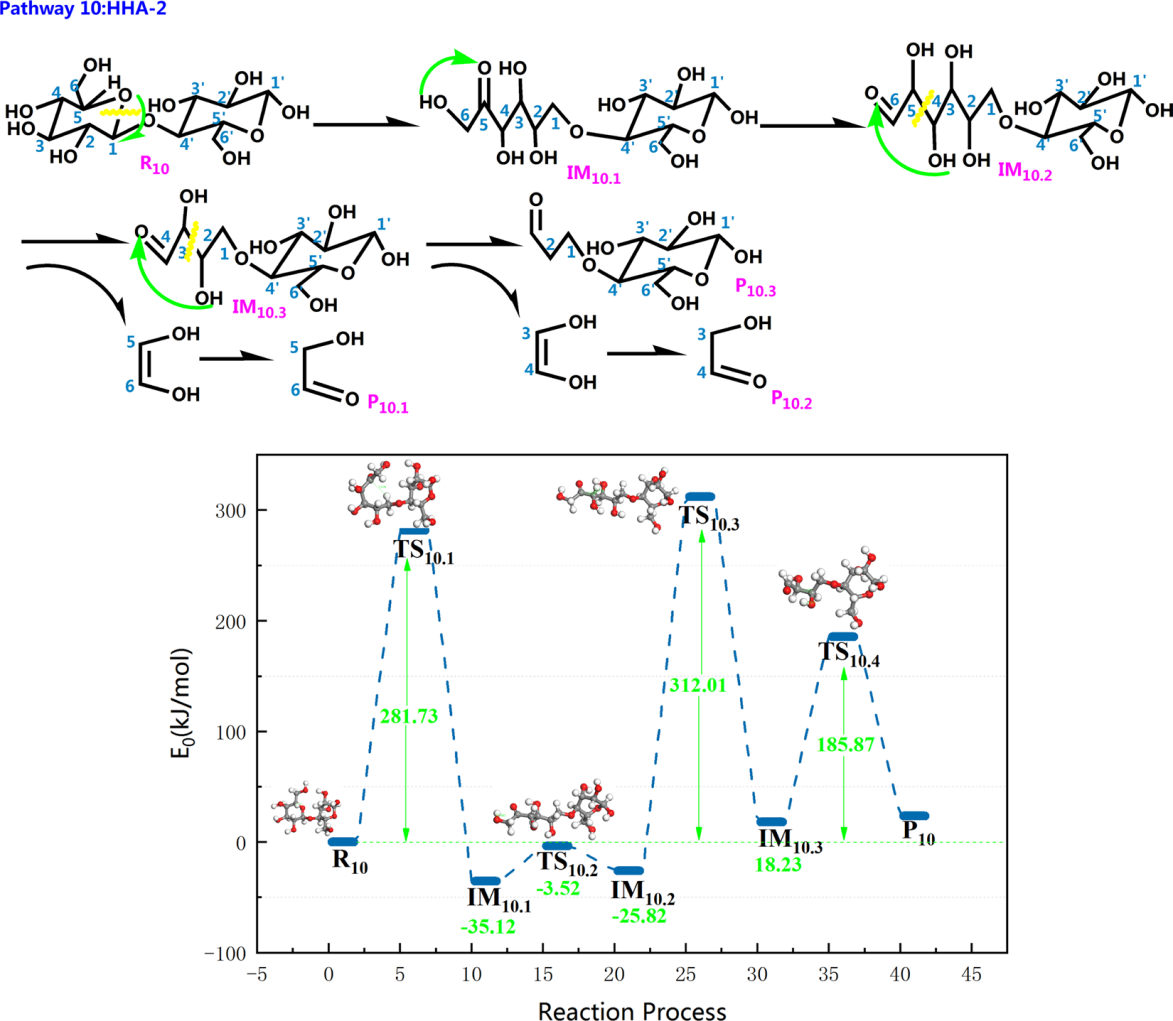
Reaction step and reaction energy barrier of pathway 10.

On the basis of previous results^62^, when the cellulose pyrolysis produces HAA molecules, cleavage of the C_1_–O bond occurs first, causing a ring opening reaction of the cellulose monomer. In the reaction step of Fig. 15, the pyran ring opens on R_10_, and the H atom is transferred from C_5_ to C_1_. Next, the H atom on O_6_–H_6_ is transferred to C_5_=O and constructs a new O_5_–H_5_ bond and a C_6_=O double bond. Intermediate IM_10.2_ is generated with a release of 3.52 kJ/mol of energy. Analogously, r(O_4_–H_4_) is 0.99 Å in intermediate IM_10.2_, and the bond length r(O_4_–H_4_) increase to 1.11 Å after the transition state TS_10.3_. Because of the longer bond length, H_4_ falls off of O_4_ and is then transferred to C_6_=O_6_. After the new double bond forms, C_4_–C_5_ cleavage releases heat of approximately 312.01 kJ/mol, and simultaneously produces a C_5_–C_6_ two-carbon small molecule fragment, which in turn undergoes a retro-diels-alder reaction to produce a small molecule of HAA. At the end of reaction process, C_2_–C_3_ breaks to grow new small molecule fragments and C_2_=O double bonds. The small molecule fragments then continue forming HAA molecules via the retro-diels-alder reaction, and thus, the barrier is as low as 185.87 kJ/mol. Analogous pathways have been proposed by previous researchers who insisted that these pathways should mainly produce HAA. The reaction energy barrier calculated by Lu *et al*.^63^ for the conversion of two carbon fragments to form HAA was 177.9 kJ/mol-391.3 kJ/mol. In contrast to the pathway 9 of HAA produced by the pyrolysis of cellobiose in this paper, the transition state TS_9.1_ was the process of cellobiose cleavaged to generate β-d-glucopyranose, absorbing 299.34 kJ/mol of heat during the reaction. Through the transition state TS_9.2_, the pyran ring of β-d-glucopyranose opened the ring to generate the small molecule product HAA and absorbed 240.75 kJ/mol of heat. This energy barrier was closer to the calculation of Lu *et al*., illustrating the rationality of the HAA generation process in pathway 10. The corresponding processes of the three transition states TS_10.1_, TS_10.2_, and TS_10.3_ of pathway 10 in this paper are the β-d-glucopyranose formation of cellobiose glycoside bond breakage, the first HAA generated, and the second HAA generated. The corresponding energy barriers of TS_10.2_ and TS_10.3_ were 312.01 kJ/mol and 185.87 kJ/mol, which were in good agreement with the calculation results of Lu *et al*. It is concluded that cellobiose has the potential to generate HAA according to the steps designed in pathway 9,10 in this paper.

**Figure 16:**
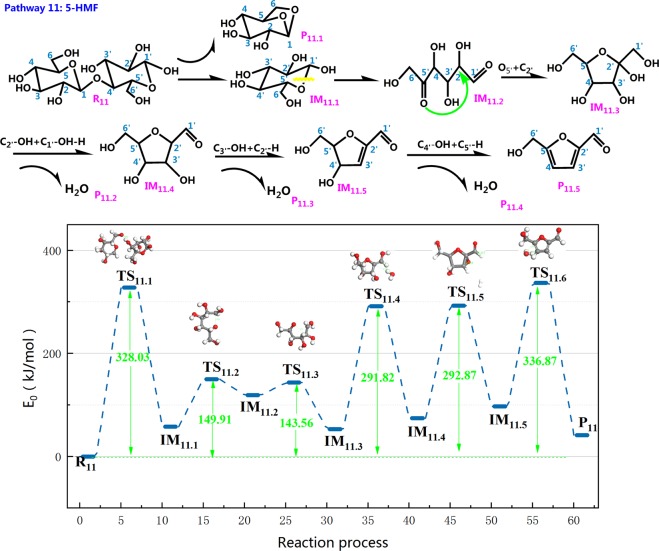
Reaction step and reaction energy barrier of pathway 11.

Throughout previous research, 5-HMF has been a significant product in the course of cellulose pyrolysis. Some scholars^64,65^ conducted a study on 5-HMF and its isomers and proposed that HMF is a dehydration derivative produced by dehydrating hexose three times. Figure 16 gives the reaction step and reaction energy barrier of pathway 11. There is a similarity between the first step in pathway 11 and pathway 1. The IM_11.2_ is generated via the rearrangement of IM_11.1_ with the C_1′_–O_5′_ of glucopyranose cleaves. Then, the new C_5_=O double bond is about to form in a ring opening reaction. Upon going through a transition state to generate a new intermediate, O_5′_ and C_2′_ in IM_11.2_ join to form a ring. The subsequent reaction procedure is three dehydration processes, and eventually the product 5-HMF is generated. Observing the energy profile of pathway 11, the energy barriers for the three dehydrations of 5-HMF were 238.65 kJ/mol, 218.4 kJ/mol, 239.6 kJ/mol, and the energy barriers for the three dehydrations calculated by Vinus^66^ were 215.58 kJ/mol, 215.58 kJ/mol, 267.90 kJ/mol, respectively. The last dehydration and cyclization occurred simultaneously, so the reaction energy barrier was higher than the other two dehydration reactions. The calculation results in this paper were close to those calculated by Vinus. It showed that pathway 11 was a possible path for cellobiose cleavage to form 5-HMF.

## Conclusion

To investigate the initial mechanism of cellulose pyrolysis, we used Py-GC/MS experiments combined with DFT theory. We explored the formation mechanism of the initial stage of cellobiose pyrolysis.

Among the three types of products produced in the thermal cracking experiment, Furan_Cellulose_ < Furan_Cellubiose_, Pyran_Cellulose_ > Pyran_Cellubiose_, and the content of small molecule products in cellobiose is higher than that in cellulose. The calculation results obtained using DFT theory are in great agreement with the experimental results. From the intrinsic theoretical research and experimental results, a modified cellulose pyrolysis model is proposed, including the formation of new reactions of LG, HAA, 5-HMF, and dehydration. From a comparison of energy barriers, it is found that of the four reaction paths for producing LG, pathway 1 is the only reaction that directly generates LG through a synergistic mechanism. Also, the energy barrier of the reaction is low, and hence, pathway 1 is the optimal pathway for generating LG. In the subsequent dehydration reaction, the reaction energy barriers are arranged in increasing order as: pathways 5, 6, 7and 8. This order indicates that the combined dehydration of C_1_–H and C_2_–OH in pathway 5 is the prime dehydration reaction during cellulose pyrolysis. Both pathways 9 and 10 are reactions in which cellulose pyrolysis produces HAA, and the HAA formation step in pathway 9 is simpler than that of pathway 10; in contrast, pathway 10 leads to higher HAA content. Finally, the reaction process of hexose to form the small molecule product 5-HMF via three dehydration steps was studied. In conclusion, the main reaction processes in the cellulose pyrolysis are pathway 1, 5, 9, and 11. This work enables further analysis of biomass energy conversion and utilization research. In addition, the pyran ring is the basic monomer structure in the long chain of cellulose. In this article, the mechanism of the pyrolysis of the pyran ring during the pyrolysis of cellulose is not sufficiently studied. In the future research, the combination of experiment and simulation will be considered to study the pyrolysis mechanism of pyran ring during cellulose pyrolysis^67,68^.

## Supplementary information

Supplementary information.
