# Supplementary material for: Initial pyrolysis mechanism and product formation of cellulose: An Experimental and Density functional theory(DFT) study
Source: Sci Rep. 2020 Feb 27;10:3626. doi: 10.1038/s41598-020-60095-2 (PMC7046763; doi:10.1038/s41598-020-60095-2)
Supplement: Supplementary file 1 — Supplementary information. [file 41598_2020_60095_MOESM1_ESM.pdf]

Initial pyrolysis mechanism and product formation of cellulose: An Experimental and Density functional theory(DFT) study

Qing Wang*, Hao Song, Shuo Pan, Nanhang Dong, Xinmin Wang, Shipeng Sun

Engineering Research Centre of Oil Shale Comprehensive Utilization, Ministry of Education, Northeast Electric Power University, Jilin City, Jilin 132012, China

* Corresponding author: Qing Wang

E-mail address: rlx888@126.com

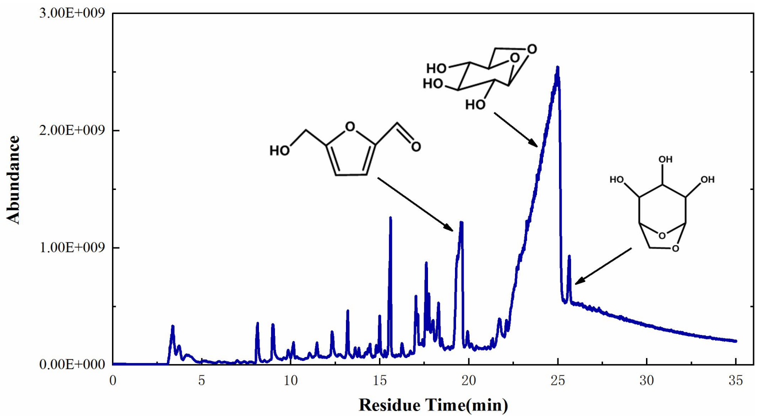

(a)

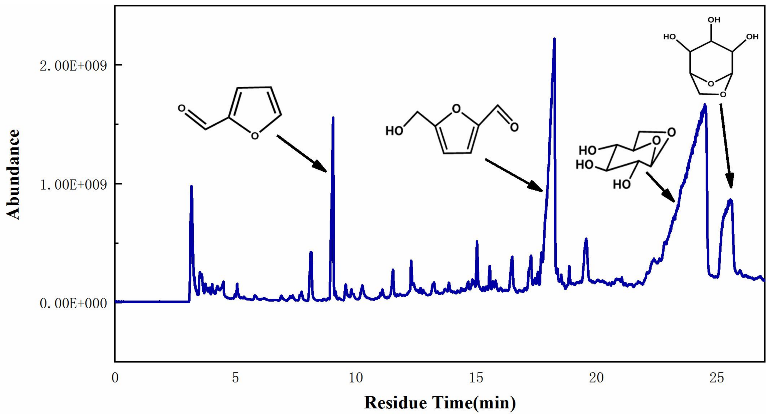

(b)

Fig. S1. Pyrolysis temperature of 400℃. Pyrolysis products identified in the pyrograms of cellulose (a),cellobiose (b) , respectively.

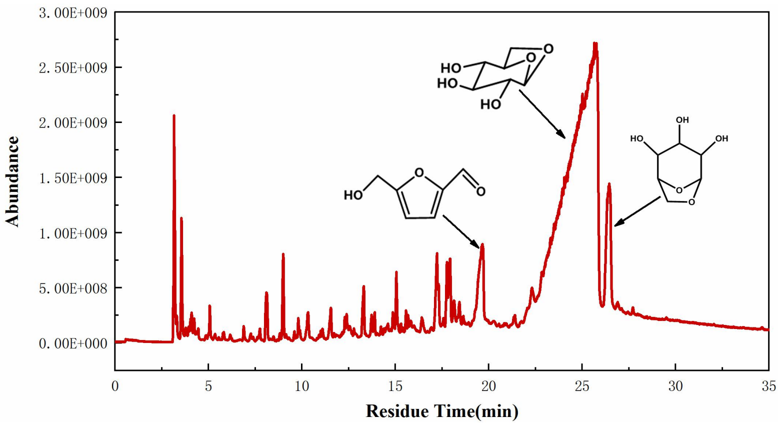

(a)

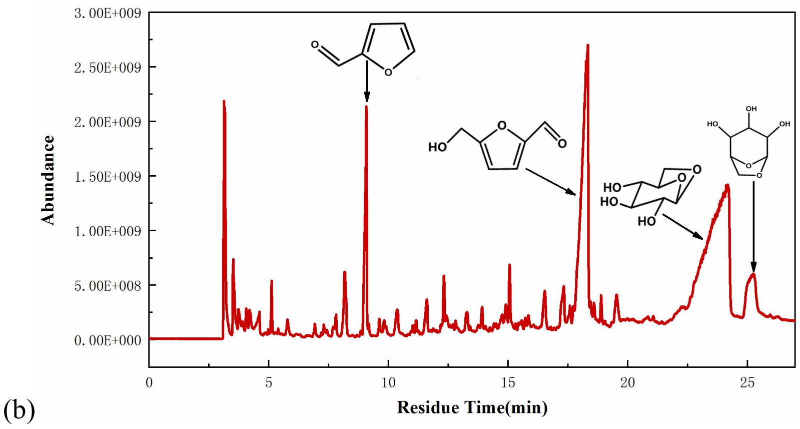

(b)

Fig. S2. Pyrolysis temperature of 500℃. Pyrolysis products identified in the pyrograms of cellulose (a),cellobiose (b) , respectively.

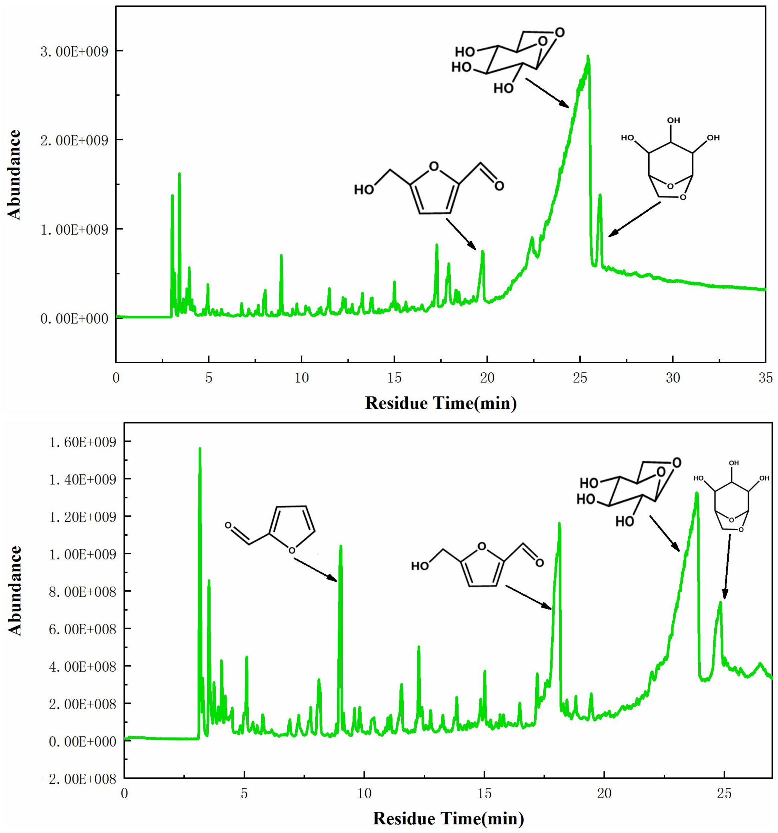

Fig. S3. Pyrolysis temperature of 600℃. Pyrolysis products identified in the pyrograms of cellulose (a),cellobiose (b) , respectively.
